# Strengthening health emergency response capacity in Kiribati: establishing the Kiribati Medical Assistance Team (KIRIMAT)

**DOI:** 10.5365/wpsar.2023.14.6.1013

**Published:** 2023-04-24

**Authors:** Sean T Casey, Anthony T Cook, May M Ferguson, Erin Noste, Katarake T Mweeka, Tabutoa Eria Rekenibai, Wendy Snowdon

**Affiliations:** a World Health Organization Regional Office for the Western Pacific, Manila, Philippines.; b School of Population Health, University of New South Wales, Sydney, New South Wales, Australia.; c World Health Organization Division of Technical Support, Suva, Fiji.; d World Health Organization Country Liaison Office for Kiribati, Tarawa, Kiribati.; e Ministry of Health & Medical Services, Tarawa, Kiribati.

The Republic of Kiribati is a small-island, large-ocean nation in the Pacific with a population of approximately 110 000. Kiribati comprises 32 low-lying coral atolls and one raised island, straddling the equator across an ocean territory of over 3.5 million km^2^ (**Fig. 1**). Given its low-lying land mass, high population density, high levels of poverty, and chronic food and water insecurity, Kiribati is particularly vulnerable to the impacts of climate change and rising sea levels. A State of Disaster was declared in June 2022 due to a severe drought. (*1*, *2*)

**Fig. 1:**
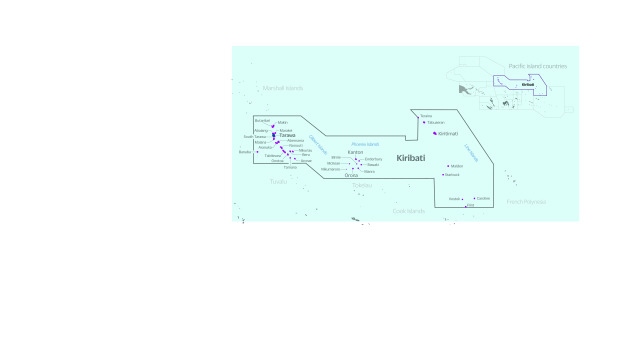
Map of Kiribati

[**insert** Figure 1]

Health services are provided by Kiribati’s Ministry of Health & Medical Services (MHMS). Across its three main archipelagos, the country’s inhabited islands are served by 115 health facilities, one national referral hospital, and a health workforce of 59 doctors and 385 nurses. (*3*) Most of Kiribati’s outer-island health facilities lack power and have limited means of communications. Many are staffed by a single nurse.

While Kiribati’s MHMS ensures continuity of essential services on all of the country’s populated islands, its capacity to mobilize surge support to outer islands when emergencies occur has in the past been hampered by a combination of transport challenges (many of Kiribati’s islands are served by infrequent passenger ships or flights), human resource constraints, and the lack of an established deployment mechanism. (*3*) Recognizing both the hazards and limitations the country faces, Kiribati’s MHMS National Health Strategic Plan for 2020–2023 sets out its intention to make health security a priority, stating that it aims to “strengthen health protection and improve community empowerment to address environmental health issues and health security including climate change, disaster risk management and outbreak control.” (*3*, *4*)

Historically, responses to outer-island emergencies in Kiribati have been mounted by ad hoc teams. In 2018, for example, an ad hoc national Emergency Medical Team (EMT) was deployed in response to a maritime disaster which left 95 dead. (*5*) Several other ad hoc clinical teams have deployed for outbreak investigations and response efforts in Kiribati’s outer islands, including most recently for the country’s COVID-19 response in 2022. However, these teams have never been formalized, and typically have lacked formal standard operating procedures (SOPs) and adequate equipment to support their deployment. To formalize this capability, the Kiribati Medical Assistance Team (KIRIMAT) was launched in November 2022 to serve as Kiribati’s deployable, self-sufficient clinical capacity for health emergency response.

## Establishing KIRIMAT

In 2019, Kiribati’s MHMS committed to establishing a national EMT capable of deploying clinical teams to all parts of the country in response to outbreaks, disasters and other emergencies with health consequences. Kiribati’s EMT was conceived with technical assistance from the World Health Organization (WHO) and with funding support from the United States Agency for International Development’s Bureau for Humanitarian Assistance (USAID/BHA). The aim of national EMT development was to enable rapid response to emergencies within Kiribati’s borders, maintaining high standards of clinical care while also ensuring the safety of both personnel and patients, in accordance with the guidance provided by WHO’s EMT handbook, “Classification and minimum standards for emergency medical teams.” (*6*) The COVID-19 pandemic and protracted border closures delayed the full development of Kiribati’s EMT for several years, but progress was made despite this challenge.

In 2021, participants from Kiribati joined a WHO-led Pacific EMT webinar series, which over a period of  11 weeks covered the core principles of EMT development and coordination. (*7*) In 2021/2022, a dedicated national EMT coordinator was hired by WHO and Kiribati’s MHMS. This role was created to support the establishment of a national EMT Technical Working Group (TWG) in Kiribati and to coordinate efforts to form the EMT, mobilizing the required resources including personnel from across the MHMS. With support from WHO and the national EMT coordinator, Kiribati’s EMT TWG drafted SOPs for KIRIMAT, based on a template designed by WHO specifically for Pacific EMTs.

Leveraging funding from USAID/BHA, WHO was able to procure equipment and supplies for KIRIMAT. This EMT “cache” comprises the equipment required by clinical and public health teams to deploy to Kiribati’s most remote outer islands, (*8*) such as generators, tents, water treatment equipment, camping gear and satellite communication equipment, as well as clinical supplies to provide emergency and outpatient care. This kit is designed to be durable and to last for at least several years and through multiple deployments. KIRIMAT and other Pacific EMTs are developing inventory management plans to ensure that caches are well maintained and in a state of readiness for rapid deployment.

KIRIMAT team members were formally inducted in November 2022, with a 5-day training session held on the island of South Tarawa involving 32 doctors, nurses, environmental health specialists, logisticians and health information specialists. The training was delivered by Kiribati’s MHMS and WHO, using a curriculum designed specifically for Pacific EMTs, with modules on clinical operations, logistics and coordination. (*9*) The training workshop culminated in a 1-day disaster response simulation exercise, which included elements of team mobilization and deployment, mass casualty triage and patient care; the simulation exercise used volunteer actors as patients and required the team to travel with their cache using a small boat.

## Discussion

With the launch of KIRIMAT in 2022, Kiribati now has in place a national EMT capable of mounting a self-sufficient national clinical and public health response to a wide range of hazards, including disease outbreaks and disasters. Low-lying and dispersed Pacific island countries and areas (PICs) such as Kiribati require this type of deployable clinical capacity to reach their most vulnerable communities in their greatest moments of need. The formal establishment of KIRIMAT, with its cohort of trained team members and a deployment-ready EMT cache tailored to Pacific contexts, means that the Kiribati MHMS will be able to reach all corners of Kiribati within hours or days of an emergency with high-quality clinical services. Kiribati is also now part of the global EMT network, a step which will continue to strengthen national response capacities and improve the coordination of international EMT support should this be required to supplement national capabilities. With the launch of KIRIMAT, Kiribati joins 12 other PICs that have established or are in the process of establishing national and international EMT capacity. (*10*) This represents a significant shift in PICs’ ability to respond to the range of hazards that they face, with increased self-reliance and capacity to serve their populations without requiring deployment of international EMTs.

## References

[1] Kiribati govt declares state of disaster due to severe drought. Radio New Zealand [Internet]; 2022. Available from: https://www.rnz.co.nz/international/pacific-news/469075/kiribati-govt-declares-state-of-disaster-due-to-severe-drought, accessed 31 December 2022.

[2] McIver L, Kim R, Woodward A, Hales S, Spickett J, Katscherian D, et al. Health impacts of climate change in Pacific Island countries: a regional assessment of vulnerabilities and adaptation priorities. Environ Health Perspect. 2016 Nov;124(11):1707–14. 10.1289/ehp.1509756 26645102 PMC5089897

[3] National Health Strategic Plan 2020–2023. Tarawa, Kiribati: Ministry of Health & Medical Services; 2020.

[4] Tassicker B, Tong T, Ribanti T, Gittus A, Griffiths B. Emergency care in Kiribati: A combined medical and nursing model for development. Emerg Med Australas. 2019 Feb;31(1):105–11. 10.1111/1742-6723.13209 30472768

[5] Harrowing details revealed of Kiribati ferry disaster that killed 95 people. News.com.au [Internet]; 2019. Available from: https://www.news.com.au/world/pacific/harrowing-details-revealed-of-kiribati-ferry-disaster-that-killed-95-people/news-story/4beb509f2e102f9a86469c2c98e1d893, accessed 31 December 2022.

[6] Classification and minimum standards for emergency medical teams. Geneva: World Health Organization; 2021. Available from: https://apps.who.int/iris/handle/10665/341857, accessed 27 January 2023.

[7] Cook AT, Devanath D, Noste EE, Beauchamin P-Y, Chandler DR, Casey ST. Adapting in-person national emergency medical teams (EMT) introductory training to a virtual, storytelling (Talanoa) format for Pacific Island countries and areas (PICs). Prehosp Disaster Med. 2022 Dec 14;37 S2:1. 10.1017/S1049023X22001893 36515181

[8] Beauchemin P-Y, Chandler DR, Noste EE, Larsen J-E, Cook AT, Casey ST. Development and procurement of a national emergency medical team (EMT) cache for Pacific island countries. Prehosp Disaster Med. 2022 Dec 15;37 S2:1. 10.1017/S1049023X22001881 36518001

[9] Casey ST, Cook AT, Chandler DR, Larsen J-E, Cowie SR, Noste EE. Tailoring and implementing an emergency medical team (EMT) training package for Pacific island countries and areas (PICs). Prehosp Disaster Med. 2022;37 S2:s95. 10.1017/S1049023X22001947

[10] Casey ST, Vatukela J, Bainivalu N, Ulufonua L, Leodoro B, Guyant P, et al. Strengthening national health emergency response: Pacific emergency medical teams (EMTs). Wkly Epidemiol Rec. 2021;96(Special Issue):iv–vii. [cited 2022 December 31] Available from https://apps.who.int/iris/handle/10665/345531

